# Determination of cervical vertebral maturation using machine learning in lateral cephalograms

**DOI:** 10.34172/joddd.41114

**Published:** 2024-12-14

**Authors:** Shahab Kavousinejad, Asghar Ebadifar, Azita Tehranchi, Farzan Zakermashhadi, Kazem Dalaie

**Affiliations:** ^1^Dentofacial Deformities Research Center, Research Institute for Dental Sciences, School of Dentistry, Shahid Beheshti University of Medical Sciences, Tehran, Iran; ^2^Department of Orthodontics, School of Dentistry, Shahid Beheshti University of Medical Sciences, Tehran, Iran; ^3^School of Dentistry, Shahid Beheshti University of Medical Sciences, Tehran, Iran

**Keywords:** Cervical vertebra dimensions, Growth modification treatment, Machine learning, Skeletal age

## Abstract

**Background.:**

The accurate timing of growth modification treatments is crucial for optimal results in orthodontics. However, traditional methods for assessing growth status, such as hand-wrist radiographs and subjective interpretation of lateral cephalograms, have limitations. This study aimed to develop a semi-automated approach using machine learning based on cervical vertebral dimensions (CVD) for determining skeletal maturation status.

**Methods.:**

A dataset comprising 980 lateral cephalograms was collected from the Department of Orthodontics, Shahid Beheshti Dental School in Tehran, Iran. Eight landmarks representing the corners of the third and fourth cervical vertebrae were selected. A ratio-based approach was employed to compute the values of C3 and C4, accompanied by the implementation of an auto_error_reduction (AER) function to enhance the accuracy of landmark selection. Linear distances and ratios were measured using the dedicated software. A novel data augmentation technique was applied to expand the dataset. Subsequently, a stacking model was developed, trained on the augmented dataset, and evaluated using a separate test set of 196 cephalograms.

**Results.:**

The proposed model achieved an accuracy of 99.49% and demonstrated a loss of 0.003 on the test set.

**Conclusion.:**

By employing feature engineering, simplified landmark selection, AER function, data augmentation, and eliminating gender and age features, a model was developed for accurate assessment of skeletal maturation for clinical applications.

## Introduction

 The timing of growth modification treatments is crucial for achieving optimal results. The peak of mandibular growth represents the ideal time for intervention.^1^ Skeletal age determination is an important method for assessing growth status in orthodontics.^2-4^ However, chronological age does not always correlate well with skeletal age,^5-7^ leading to the introduction of alternative methods for skeletal age assessment.^8-11^ While hand-wrist radiographs are considered the gold standard for skeletal age determination,^12^ their limited use in dentistry is due to concerns about excessive radiation exposure.^13-15^ In dentistry, evaluating cervical vertebra maturation (CVM) on lateral cephalograms is the most common approach to assessing skeletal age, as it is easy to perform and provides valuable information for initial diagnosis in orthodontics.^10,16-18^

 However, interpreting lateral cephalograms for CVM analysis can be challenging due to variations in image clarity and the absence of a definitive cutoff point between CVM stages.^18,19^ By incorporating a quantitative approach, we can enhance our understanding of the patient’s skeletal maturation, ultimately leading to more effective treatment outcomes. Moreover, several studies have reported low inter- and intra-observer agreement, indicating that the CVM method lacks reliability and reproducibility.^1,20-23^ These limitations arise from the qualitative nature of parameters assessed in the CVM method, such as the amount of concavity and the shape of cervical vertebrae, highlighting the need for quantitative approaches. Quantitative methods have been developed to address these limitations, focusing on measuring cervical vertebra dimensions (CVD) to determine skeletal age.^9,24,25^ A strong and statistically significant correlation between CVM and CVD has been demonstrated.^25^ In this method, the six groups (CVM method) were divided into three groups. Groups 3 and 4 in the CVM method (group 2 in the CVD method) are associated with the mandibular peak growth period ^25,26^ Therefore, we used the three-class method (pre-peak, peak, and post-peak) in the present study.

 In recent years, the rapid advancement of imaging technologies, coupled with the increasing complexity of interpretation, has sparked a surge of interest among researchers in exploring the potential application of artificial intelligence (AI) in orthodontics. AI can potentially assist orthodontists in diagnosing and predicting outcomes with high accuracy and reduce time compared to trained dentists.^27^ Several studies have evaluated the accuracy of deep learning models in determining CVM stages. Atici et al^28^ and Khazaei et al^29^ achieved accuracy rates ranging from 75% to 82%. Kök et al^30^ compared deep learning models with machine learning models and concluded that deep learning models outperformed machine learning models. However, considering the novel methodology employed to measure CVD in the present study, integrating feature engineering and feature selection into machine learning models is expected to yield significantly higher accuracy than deep learning models. This study aimed to determine skeletal maturation status using machine learning algorithms based on quantitative measurements of CVD obtained from lateral cephalograms. By leveraging the potential of AI, this research aimed to enhance the accuracy of skeletal age assessment in orthodontics.

## Methods

###  Data collection and dataset preparation

**Figure 1:**
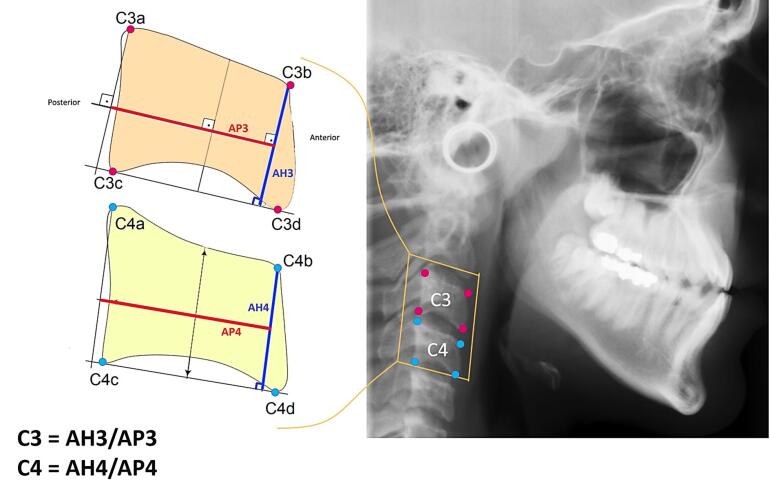
The selected landmarks, indicated by red and blue dots, represent the corners of the third and fourth cervical vertebrae, respectively. Eight landmarks were selected. For C4, a red line connects the midpoint of the perpendicular line from C4a to the line C4c-C4d with the midpoint of the perpendicular line from C4b to the line C4c-C4d. The length of this line represents the value of AP4. The blue line, AH4, shows the perpendicular line from C4b to the line C4c-C4d. The value of C4 is presented as the ratio AH4/AP4. A similar method was applied to the third cervical vertebra, determining the value corresponding to C3.


 In this study, 980 digital cephalograms were collected from 6‒17-year-olds. The cephalograms were collected from existing files in the Department of Orthodontics, Shahid Beheshti Dental School, Tehran, Iran. Inclusion criteria consisted of high-quality cephalograms and cervical vertebrae and the absence of specific syndromes and systemic problems in patients. Each cephalogram was randomly assigned a unique identifier in the format of a letter and a value (e.g., A0). To perform feature engineering, the ratio of CVD was calculated according to the following formula.^25^ This method is described in Figure 1.

$$C_{3}=\frac{AH_{3}}{AP_{3}},\;C_{4}=\frac{AH_{4}}{AP_{4}}$$

In this method, eight landmarks representing the corners of the third and fourth cervical vertebrae were meticulously selected. In cases where the corners exhibited curvature, the midpoint of the curve was selected. A software application was developed using the C# programming language to facilitate the measurement of linear distances and ratios. The cephalograms were subsequently imported into the software, where they underwent resizing to achieve a uniform width of 2000 pixels while preserving the original aspect ratio. This resizing operation was necessary to standardize the pixels for subsequent steps. To calculate the lengths, the pixel count between the selected landmarks (X and Y coordinates) was measured using the following formula:


$$Length=\sqrt{{\left(x_{2}-x_{1}\right)}^{2}+{\left(y_{2}-y_{1}\right)}^{2}}$$

**Figure 2:**
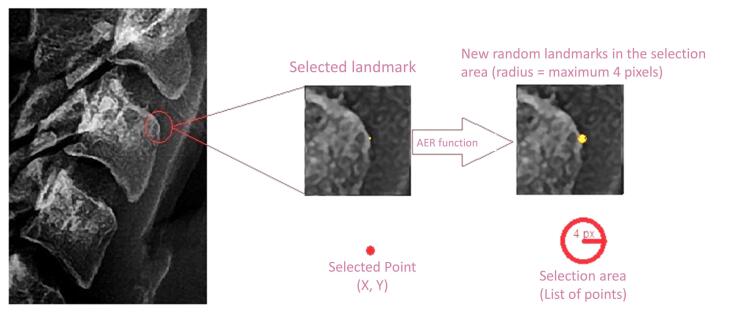
The AER function’s operational procedure is depicted. We anticipated an operator error of up to 4 pixels during point selection, which the function systematically addresses. Within this function, a thousand randomly generated points are automatically distributed across the designated selection area. The corresponding values of C3 and C4 were calculated for each generated point. This iterative process is repeated a thousand times, resulting in a dataset of a thousand C3 and C4 values for each sample. Finally, the average values of C3 and C4 were computed and considered as the definitive C3 and C4 values for each sample, respectively.

The values of C3 and C4 were calculated for each sample. By employing the ratio-based approach, inherent variations in magnification associated with diverse radiographic views and x-ray devices were effectively eliminated. An innovative AER function was implemented within the software framework to enhance the accuracy of landmark selection by the researcher (Figure 2). Within this function, the coordinates of each selected landmark within the software were subjected to random displacements spanning 1 to 4 pixels in both the X and Y directions relative to the original landmark. Subsequently, the values of C3 and C4 were computed for each iteration of the AER function, which was repeated a thousand times (as a loop). Ultimately, the average values of C3 and C4 were derived as the output for each sample. This sophisticated approach substantially reduced the error stemming from discrepancies in landmark selection across researcher iterations, as the selection area encompassed a set of randomly distributed landmarks within a maximum radius of 4 pixels. AER function is a probabilistic average of surrounding landmarks.

 The data, including age, gender, C3 and C4 values, were placed in a CSV file. A three-class label column called “Maturation” was considered in this CSV file. To prevent bias, blind labeling was performed, meaning the expert determining the class of each sample was unaware of the features of each sample. Initially, labeling was done based on the CVM method for each sample’s cephalometric measurements by an orthodontist. Using this method, six classes were identified (CVS1 to CVS6). In the next step, the final classification (maturation) was determined as follows:

 Class 1: Pre-peak of mandibular growth (CVS1 and CVS2 classes)

 Class 2: Peak of mandibular growth (CVS3 and CVS4 classes)

 Class 3: Post-peak of mandibular growth (CVS5 and CVS6 classes)

 Two other important indices, SumC3C4 and C3C4, were calculated for each sample based on the following formulas and included in the dataset:


$$\begin{array}{l} SumC3C4=C_{3}+C_{4}\\ C_{3}C_{4}=C_{3}\times C_{4} \end{array}$$

Therefore, the final dataset included age, gender, C3, C4, SumC3C4, C3C4, and maturation. The project was coded in Python programming language using the Jupyter Notebook environment (version 6.4.12). The following Python libraries were used: Scikit-learn (sklearn), CatBoost, LightGBM, and XGBoost.

###  Data preprocessing and feature selection

**Figure 3:**
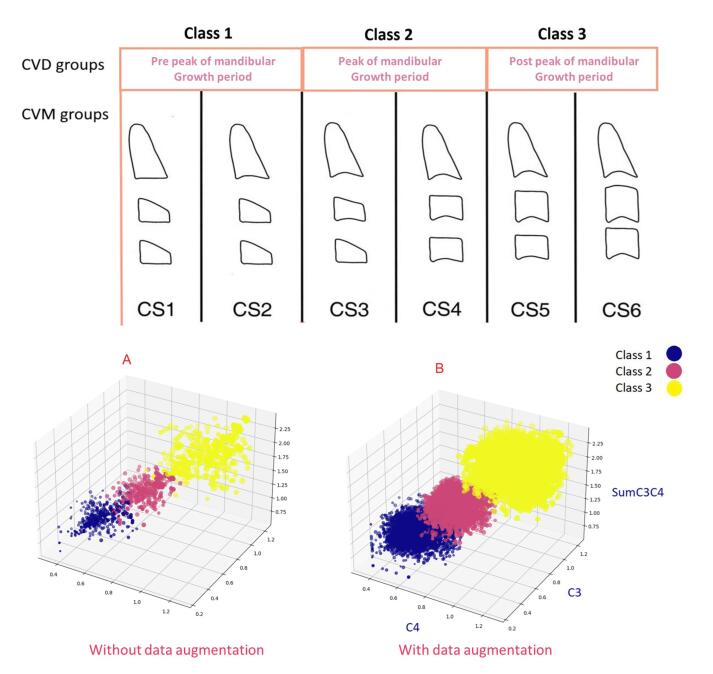
Visualization of the training dataset in 3D format before (A) and after (B) data augmentation

 The dataset had no missing values, and the sample sizes for each class were balanced. The labels were converted from qualitative (pre-peak, peak, post-peak) to quantitative, representing three classes: 1, 2, and 3. The dataset was randomly split into two sets: train (80%) and test (20%). Figure 3 visually presents the three-dimensional distribution of the training data categorized by class before and after data augmentation. The x, y, and z axes correspond to the values C3, C4, and SumC3C4, respectively. Additionally, the size of each sample (represented as a sphere) is determined by the weighted value of C3C4.

 To enhance the generalization and accuracy of our machine learning model, introduce data diversity, and mitigate overfitting, we developed a data augmentation technique called CVD_Generator specifically for the training data. This method involves generating random values within the range of minimum and maximum values for C3 and C4 for each class, considering their distributions within the training data. As a result, 1000 new samples were created for each class, with random values of C3 and C4 based on their respective classifications. Additionally, the values of SumC3C4 and C3C4 were calculated for each newly generated sample. The CVD_Generator method is defined as follows:

 n = 1000, G = {1, 2, 3}, C = {C3, C4}

 Where n represents the number of new samples, and G is the set of groups defined based on the maturation column. The values in C include the set of columns C3 and C4. For each group gi ∈ G and each value j ∈ {1, 2,..., n}, a new sample was generated as follows:


$$S_{ij}=\left\{\begin{array}{l} C_{3}:v_{ij3},\;C_{4}:v_{ij4},\;SumC3C4:v_{ij3}+v_{ij4},\\ C_{3}C_{4}:v_{ij3}\times v_{ij4},\;Maturation:g_{i} \end{array}\right\}$$

Where S_ij_ is a new row added to the data frame, and v_ijk_ is a new random value of the C_k_ column within the range of values for that group. Then, the age and gender values were also randomly generated based on their respective distributions in each class for the new samples.

 To reduce the model’s reliance on age and gender and mitigate bias, we exclusively focused on the independent variables (X): C3, C4, SumC3C4, and C3C4, while considering the three-class label as the dependent variable (Y). As the selected features possess the same nature (dimension ratio), we refrained from applying any data normalization techniques (e.g., employing the StandardScaler method) or feature scaling to them.

###  Model architecture

 Figure S1 visually represents the data preprocessing process, model architecture, and model testing. In the initial stage, we employed the 5-fold cross-validation technique along with grid search and genetic algorithms (for MLP) to determine the optimal hyperparameters for each stage 1 model. The hyperparameters of each model were adjusted accordingly. Subsequently, each model underwent individual training using the training data and evaluation using the test data. Consequently, the hyperparameters for the base models were appropriately configured. The fine-tuned models were then integrated as base models within the stacking model (depicted as stage 1 in Figure S1). The stacking model was trained using the training data. The fundamental concept of model stacking involves training multiple diverse base models and combining their predictions through the training of a meta model. The meta model generates the final prediction by considering the predictions made by the base models. The base models underwent training based on 5-fold cross-validation (CV) on the data and forwarded their predictions to the final estimator. In our proposed model, we used logistic regression as the final estimator, employing default hyperparameters. The 5-fold CV technique divides the data into 5 subsets, using one-fifth as the test data and the remaining 4 subsets as the training data in each iteration. The final prediction is then obtained by averaging the results of these 5 iterations. The architecture of the stacking model is depicted in Figure S1.

###  Base models

- ExtraTreesClassifier
- Multi-layer perceptron (MLP)
- XGBClassifier
- CatBoostClassifier
- LGBMClassifier (LightGBM)
- VotingClassifier

###  Final model

- Classification Meta Model: As the ultimate estimator in the stacking model, logistic regression was used, providing a reliable and interpretable prediction for the ensemble model.

###  Evaluation

 In evaluating our model on the test data, we employed various metrics to assess its effectiveness. These metrics encompass accuracy, precision, F1 score, recall, log loss, Jaccard, and the confusion matrix.

**Log loss:** This function gauges the performance of a classification model by computing the negative logarithm of the predicted probability for the correct label. In the provided formula, N denotes the number of samples, *y*_ij_ represents the true label for sample *i* and class *j*, and *p*_ij_ corresponds to the predicted probability for sample *i* and class *j*.


$$L\left(y,p\right)=-\frac{1}{N}\sum_{i=1}^{N}\sum_{j=1}^{3}y_{ij}\log\left(p_{ij}\right)$$

**Accuracy:** Accuracy measures the overall correctness of the model’s predictions, calculated as the ratio of correct predictions to the total number of predictions.


$$accuracy=\frac{TP+TN}{TP+TN+FP+FN}$$

- TP (True Positive): The number of positive instances correctly predicted as positive
- FP (False Positive): The number of negative instances incorrectly predicted as positive
- TN (True Negative): The number of negative instances correctly predicted as negative
- FN (False Negative): The number of positive instances incorrectly predicted as negative

**Precision:** Precision assesses the model’s capability to accurately predict positive samples, computed as the ratio of true positive predictions to the total number of predicted positives.


$$precision=\frac{TP}{TP+FP}$$

**Recall:** Recall evaluates the model’s ability to correctly identify all positive samples, expressed as the ratio of true positive predictions to the total number of true positives.


$$recall=\frac{TP}{TP+FN}$$

**F1 score:** The F1 score represents a measure of the balance between precision and recall, computed as the harmonic mean of precision and recall.


$$F1score=2\times \frac{precision\times recall}{precision+recall}$$

Additionally, as an extra objective in our study, we used the following formula to determine the cutoff points between the three classes:

 Class_1_range = {x∈X∣y(x) = 1}, Class_2_range = {x∈X∣y(x) = 2}, Class_3_range = {x∈X∣y(x) = 3}


$$cut\_point\_1=\frac{\max\left(Class\_1\_range\right)+\min\left(Class\_2\_range\right)}{2}\;$$

The first and second cutoff points were determined using the following formula.


$$cut\_point\_2=\frac{\max\left(Class\_2\_range\right)+\min\left(Class\_3\_range\right)}{2}\;$$

To ensure the presence of a cutoff point that includes both the SumC3C4 and C3C4 features, we devised the following formula:


$$\begin{array}{l} Final\;cuttoff\;point\_1\;=\frac{cuttoff\_1\;\left(C3C4\right)}{cuttoff\_1\;\left(SumC3C4\right)\;}\times 100\\ Final\;cuttoff\;point\_2=\frac{cuttoff\_2\;\left(C3C4\right)}{cuttoff\_2\;\left(SumC3C4\right)\;}\times \text{100 } \end{array}$$

## Results

**Figure 4:**
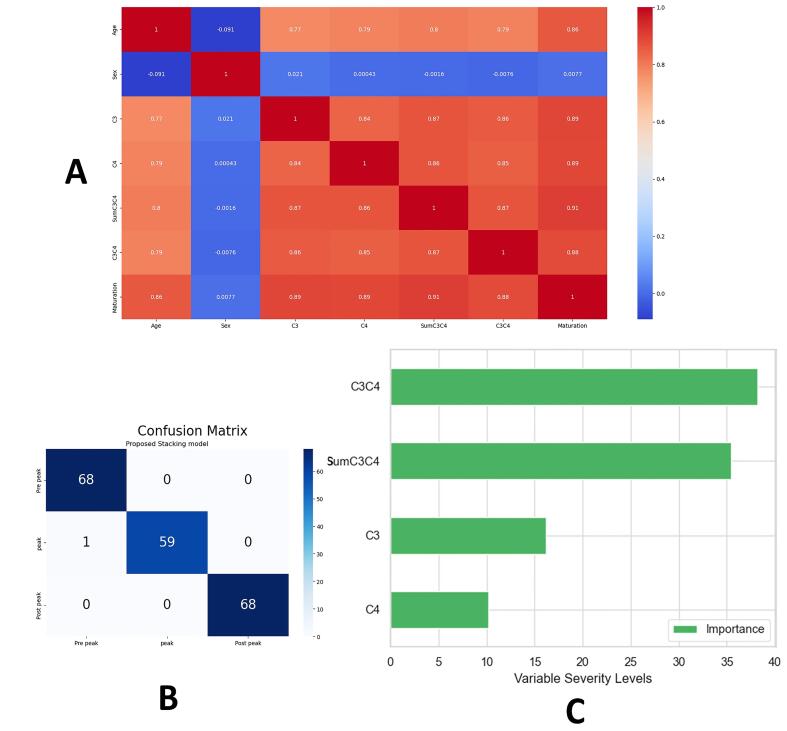
A) Heatmap of the correlation among the initial dataset features. B) Confusion matrix of the proposed model on the test data. C) The importance of each feature for the proposed model

Table 1 shows the means and standard deviations of dataset features. Figure 4a visualizes the correlation among the initial features, indicating a weak correlation with “gender” and strong positive correlations among the other features. Table 2 presents the results of fine-tuning various base models, with the stacking model outperforming base models (99.49% accuracy, 0.003 log loss). The confusion matrix for the proposed model on the test data is shown in Figure 4b. Sensitivity analysis in Figure 4c highlights the highest importance of “C3C4” and the least importance of “C4.” Table 3 displays cutoff points between the three groups based on “SumC3C4” and “C3C4” features. The dataset ranges from a minimum SumC3C4 value of 0.62 to a maximum of 2.38 and a minimum C3C4 value of 0.09 to a maximum of 1.42.

**Table 1:** The mean and standard deviation for C3C4, SumC3C4, C4, C3, and age in the initial dataset

**Maturation**	**Age** **(mean±SD)**	**Gender** **(Counts)**	**C3** **(mean±SD)**	**C4** **(mean±SD)**	**SumC3C4** **(mean±SD)**	**C3C4** **(mean±SD)**
Pre peak (n = 326)	8.61 ± 1.65	M:192 F:134	0.52 ± 0.08	0.49 ± 0.08	1.01 ± 0.14	0.26 ± 0.07
Peak (n = 326)	11.53 ± 1.37	M:178 F:148	0.74 ± 0.08	0.68 ± 0.08	1.42 ± 0.14	0.51 ± 0.1
Post peak (n = 328)	16.1 ± 1.23	M:194 F:134	1.01 ± 0.14	0.94 ± 0.12	1.96 ± 0.23	0.97 ± 0.23

**Table 2:** Model performance metrics on the test data and fine-tuned hyperparameters for base models

	**Model**	**Accuracy (%)**	Precision*	Recall*	F1 score*	**Log loss**	**Hyperparameters (fine-tuned)**
Base models	Support vector machine	96.94	0.97	0.97	0.97	0.08	Kernel = 'rbf', C = 800
	K-nearest neighbors	97.96	0.98	0.98	0.98	0.19	n_neighbors = 4
	Random forest	98.98	0.99	0.99	0.99	0.06	n_estimators = 211, max_depth = 13
	Extra trees classifier	97.44	0.98	0.97	0.97	0.10	n_estimators = 63, max_depth = 12
	Multi-layer perceptron	97.45	0.98	0.97	0.97	0.09	hidden_layer_sizes = (20,), learning_rate_init = 0.01 (adaptive), activation = logistic, max_iter = 300, solver = 'adam', alpha = 0.0001
	XGB classifier	98.98	0.99	0.99	0.99	0.02	n_estimators = 100, max_depth = 3, learning_rate = 0.2, subsample = 0.9, colsample_bytree = 0.85
	CatBoost classifier	97.42	0.98	0.98	0.98	0.09	Iterations = 100, learning_rate = 0.1, depth = 5
	LGBM classifier	98.98	0.99	0.99	0.99	0.04	n_estimators = 1000, learning_rate = 0.1, max_depth = 10
	Voting classifier	98.47	0.99	0.98	0.98	0.09	Default
Final model	Proposed Stacking model	**99.49**	1.0	0.99	0.99	0.003	final_estimator (logisticRegression)

* Weighted average

**Table 3:** The cutoff points for class separation based on C3C4 and SumC3C4 features

**Class separation**	**Cutoff point SumC3C4**	**Cutoff point C3C4**	**Final cutoff point**
Between Class 1 and Class 2	1.22	0.365	29.91
Between Class 2 and Class 3	1.56	0.62	39.74

## Discussion

 The present study aimed to assess skeletal maturation using cervical vertebrae. We gained valuable insights into feature engineering, correlation visualization, model fine-tuning, sensitivity analysis, and classification cutoff points through data analysis and model evaluation. The proposed model achieved 99.49% accuracy and a test set loss of 0.003, outperforming base models. This highlights the effectiveness of combining multiple models to improve skeletal maturation prediction accuracy.

 The relationship between hand-wrist radiographs and skeletal age is well established.^10,31,32^ Kim et al^33^ found that an ensemble model of eight machine learning models achieved the highest accuracy of 43% in predicting hand-wrist maturation stages based on cervical vertebrae from lateral cephalograms. However, the CVM method on lateral cephalograms is widely recognized as a reliable approach for determining skeletal age.^16^

 Two studies compared the performance of deep learning models with human visual analysis and reported a low agreement percentage of 58%, possibly due to small sample sizes or the specific AI models used.^34,35^ Khazaei et al^29^ increased the sample size to 1846 patients in a study using CNN models, resulting in a higher agreement percentage. However, the highest accuracy was still relatively low in the three-group classification at 82%. Similarly, Atici et al^28^ used data augmentation and found their CNN model superior to other deep learning models, but the accuracy remained below 83% for females and 75% for males. In contrast, Seo et al^36^ achieved a higher average accuracy of 95.6% using a deep learning approach and image segmentation on a relatively large sample size of 900 participants for bone age estimation based on the CVM method. Therefore, one strength of our study is the use of a large sample size and a novel data augmentation approach.

 What sets our study apart is its higher accuracy, using only eight vertebral reference points and four linear measurements. In contrast, Amasya et al^37^ compared five machine learning models in CVM analysis using 26 marked landmarks and evaluating 54 features on each lateral cephalogram, and their results indicated that ANN had the highest agreement of 86.93% with visual analysis. Additionally, Xie et al^38,39^ achieved accuracies of 87% and 88% in two separate studies by considering various parameters such as chronological age, C3 height (H3), and the ratio of posterior height to lower width of C4 (PH4/LW4). Kök et al^40^ evaluated 24 ANN models with 27 vertebral reference points and 32 linear measurements, with the best model achieving an accuracy of 94.27% using 32 linear measurements and age. The highest accuracy with the fewest linear measurements (13) was 86.87%. Therefore, the advantages of our study include higher accuracy, fewer landmarks, AER function, data augmentation, and feature engineering.

 The results revealed a weak correlation between the “gender” variable and other features, while strong positive correlations were observed among the remaining features. These findings suggest that the “gender” variable may have limited influence on skeletal maturation assessment, while the other features exhibit interdependencies that can be leveraged for accurate evaluation. In this study, we aimed to develop a skeletal maturation assessment model free from gender and age bias. To achieve this, we excluded the gender feature from the model input, as it showed no significant correlation with other factors. Additionally, we removed the age feature to ensure that our model solely relies on the geometric dimensions of the third and fourth vertebrae. Consequently, when the model is deployed in the application software, we may confidently avoid the influence of chronological age on skeletal maturation status, even if an individual presents with a higher chronologic age but has delayed skeletal maturation due to factors such as illness, syndrome, or vitamin D deficiency.^41^

 We used feature engineering and machine learning techniques to evaluate skeletal maturation based on cervical vertebrae. We focused on the changing dimensions of the third and fourth vertebrae as important features through feature engineering, which may explain the lower accuracies observed in CNN studies. By considering the variability of the anterior border of the third and fourth vertebrae during the 6-stage cervical vertebral maturation process, we emphasized the length features of AH3 and AH4. To standardize radiographs, we used ratios by dividing these values by AP3 and AP4. Consequently, the values of C3 and C4 contain valuable information about skeletal growth features. We also introduced the features SumC3C4 and C3C4 to represent an individual’s current peak growth status within a specified range. C3C4 had the most significant impact on the classification model among these features. Multiplying features together may increase their importance, suggesting the advantage of generating new features by combining existing ones in other machine learning studies with numerous features in this field.

 This study introduces a new method for selecting cervical vertebra landmarks, using a simplified process and fewer landmarks. Instead of using many landmarks, we only selected eight landmarks from the cervical vertebrae on lateral cephalometric radiographs, making the process faster and more user-friendly. This approach offers a more efficient alternative to previous machine learning studies on cervical vertebrae. The core of our proposed method is the AER function, which reduces researcher error in landmark selection. We predicted a four-pixel operator error within the AER function. We standardized the width of all cephalometric images while maintaining the aspect ratio and performed landmark selection three times on 20 randomly selected samples. On average, the coordinates of three points for each landmark fell within a four-pixel radius. By automatically executing the AER function, we calculated the values of C3 and C4 a thousand times for each sample. This procedure improved calculation accuracy, reduced bias, and minimized landmark selection errors.

 By maintaining the data distribution, our data augmentation approach effectively generated additional samples, enhancing the diversity present in the data. This approach helped avoid overfitting, enhancing the model’s ability to generalize. Additionally, by increasing the quantity of training data within each class, our augmentation method provided the model with more samples to learn from patterns.

 Unlike previous studies, our method eliminated the need to determine the curvature of the inferior border and focused on the correlation between vertebral dimensions and stages of CVM. The classification model used in the study did not rely on chronological age, enhancing confidence in the results’ validity and accuracy. The study suggested that the optimal timing for growth modification in the CVM method is between CS3 and CS4. However, according to the three-class cervical vertebral maturation method, the middle of group 2 is considered the best treatment timing. This implies that the patient’s current skeletal position can be visually represented as resembling Figure S2.

## Conclusion

 The proposed model achieved an accuracy of 99.49% in evaluating skeletal maturation based on cervical vertebrae. Overall, by employing feature engineering, simplified landmark selection, AER function, data augmentation, and the elimination of gender and age features, a model has been developed for accurate assessment of skeletal maturation for clinical applications.

## Acknowledgments

 We would like to thank the Dentofacial Deformities Research Center at Shahid Beheshti University of Medical Sciences for their support and contributions to this research. We thank the faculty members, researchers, and staff for their guidance and expertise.

## Competing Interests

 None.

## Ethical Approval

 This study was approved by Shahid Beheshti University of Medical Sciences (IR.SBMU.DRC.REC.1402.126).

## Supplementary File

Supplementary file contain Figures S1 and S2.
