# Supplementary material for: Determination of cervical vertebral maturation using machine learning in lateral cephalograms
Source: J Dent Res Dent Clin Dent Prospects. 2024 Dec 14;18(4):232–41. doi: 10.34172/joddd.41114 (PMC11786010; doi:10.34172/joddd.41114)
Supplement: Supplementary file 1 — Supplementary file contain Figures S1 and S2. [file joddd-18-232-s001.pdf]

Kavousinejad et al, ***J Dent Res Dent Clin Dent Prospects,*** 2024, 18(4), 232-S1
doi: 10.34172/joddd.41114
https://joddd.tbzmed.ac.ir

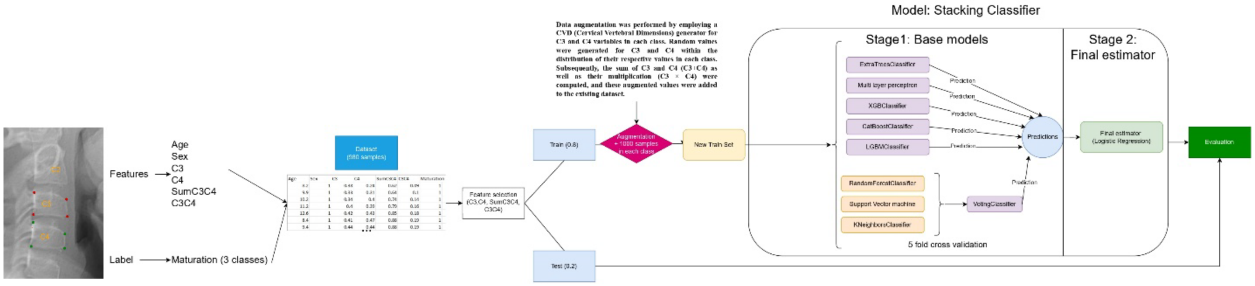


**Figure S1.** Visual representation of the data preprocessing and model architecture

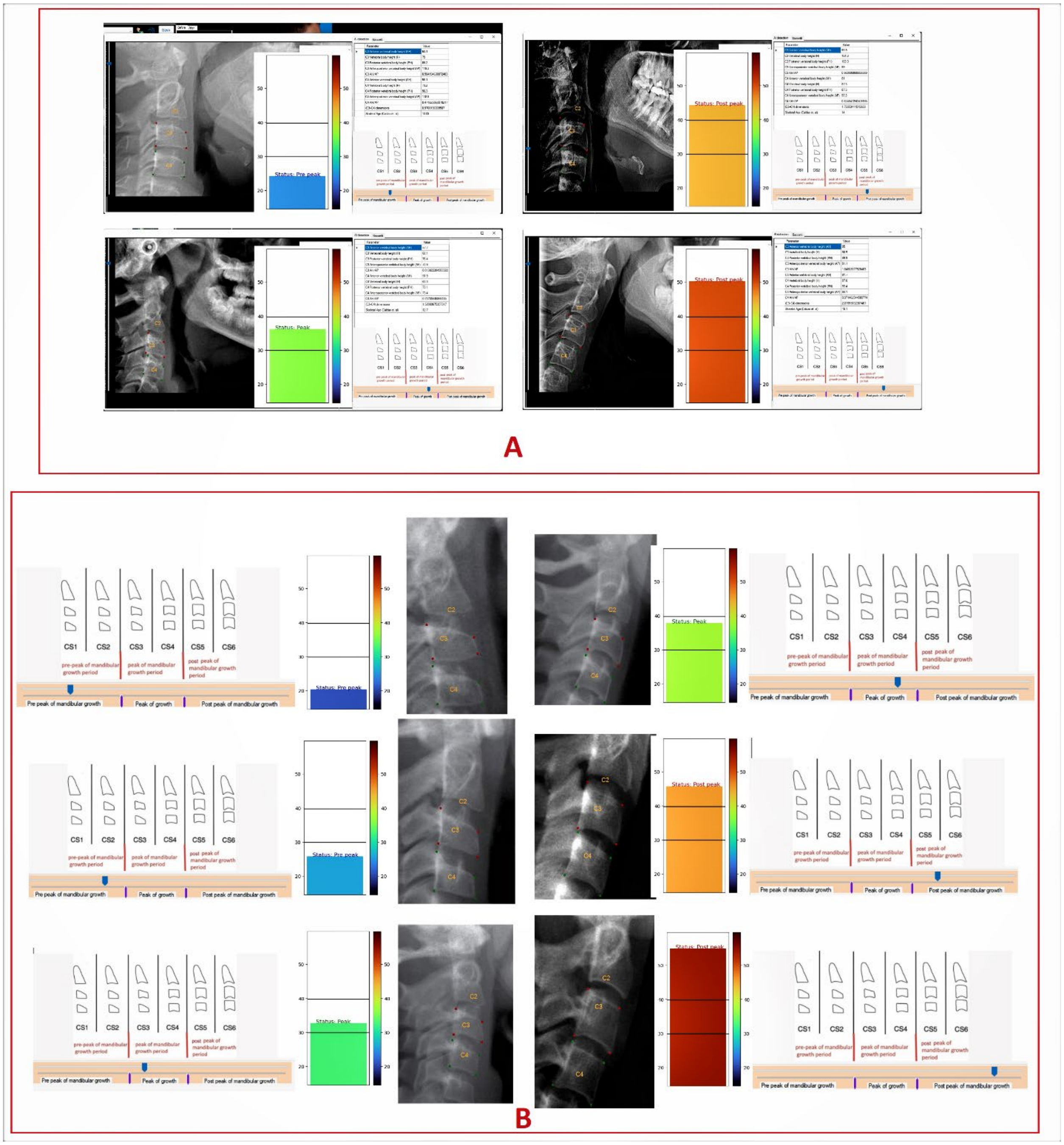


**Figure S2.** Part A displays four test data samples that are inputted into the C#-based software where our model was deployed. By selecting the landmarks, the user uses the software (the deployed model) to predict the patient's growth status. Part B illustrates each stage of CVM predicted by the model. According to the method in this study, if the patient's growth status falls between the two threshold values, it is represented by green. When the growth status color is green, it is the optimal time for prescribing functional class 2 appliances. As the color shifts towards yellow and red, the effectiveness of the functional appliance diminishes. The "status" in the color graphs represents the model's prediction, and the bar and color grading in the graphical representation are based on the formula "$\text{Patient} = \frac{\text{C3C4}}{\text{SumC3C4}} \times 100$". The black lines in the color graphs indicate the cutoff points
